# Integration of Genome‐Skimming Sequencing and Morphological Evidence Reveals Two New Endemic Species of *Sinocrassula* From Yunnan Province, China

**DOI:** 10.1002/ece3.73465

**Published:** 2026-05-04

**Authors:** Jing Zhao, Ling‐Nan Wei, Jie Zhou, Jian‐Rong Zhang, Xin‐Mao Zhou, Zhao‐Rong He, Chao Chen, Jia‐Guan Wang

**Affiliations:** ^1^ School of Life Sciences & School of Ecology and Environmental Science Yunnan University Kunming Yunnan China; ^2^ State Key Laboratory of Vegetation Structure, Functions and Construction Yunnan University Kunming Yunnan China; ^3^ Yunnan Key Laboratory of Forest Ecosystem Stability and Global Change, Xishuangbanna Tropical Botanical Garden, Chinese Academy of Sciences Mengla Yunnan China; ^4^ Laboratory of Tropical Forest Ecology, Xishuangbanna Tropical Botanical Garden, Chinese Academy of Sciences Mengla Yunnan China; ^5^ Yuanjiang Savanna Ecosystem Research Station, Xishuangbanna Tropical Botanical Garden, Chinese Academy of Sciences Yuanjiang Yunnan China

**Keywords:** crassulaceae, new species, nuclear genes, taxonomy, Yunnan province

## Abstract

*Sinocrassula* represents the medium‐sized Asian genus within Crassulaceae, exhibiting maximum species diversity in China. This study presented the first comprehensive phylogenetic analyses of *Sinocrassula*, utilizing both sanger data and next‐generation sequencing data. We confirm that three populations of *Sinocrassula* from Yunnan Province represent two distinct new species, and we described them as *S. crassifolia* Chao Chen, Jing Zhao & J. Guan Wang (including two populations) and *S*. *yongshengensis* Chao Chen & J. Guan Wang (one population). Morphologically, these two new species resemble *S. indica* var. *obtusifolia*. However, phylogenetic analyses place these two new species closer to *S*. *adpressa*. According to IUCN guidelines, the two new species are preliminarily assessed as “Data Deficient (DD).” The findings hold significant implications for understanding the flora of the Himalaya‐Hengduan Mountains (HHM) region and lay the groundwork for future development of ornamental and medicinal resources in the family of Crassulaceae.

## Graphical abstract

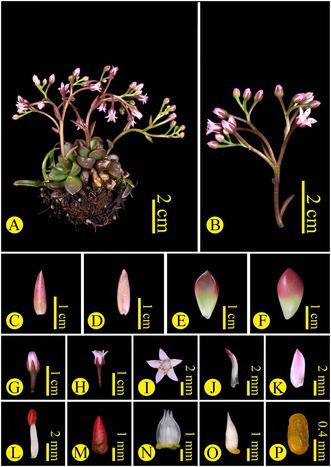

## 1 Introduction

*Sinocrassula*, established by Berger (1930) based on *Sinocrassula indica* (Decne.) A. Berger, is a medium‐sized lineage of succulent plants (T'Hart 1995; Eggli 2005; Zhao et al. 2025). *Sinocrassula* is distributed primarily in Asia, particularly in the Himalaya‐Hengduan Mountains (HHM) (Fu and Ohba 2001; Zhao et al. 2025). However, the diversity of the genus has been severely underestimated during the past nearly 100 years. Before 2000, it was generally accepted that *Sinocrassula* comprised only seven species and five varieties of *S. indica* (Fu and Ohba 2001). In recent years, with the advancement of phylogenetic inferences and increased attention from Chinese scholars, amount of morphologically distinct species of *Sinocrassula* supported also by molecular evidence have been published, nearly doubling the number of species (ca. 16 species and five varieties) within the genus (Wang et al. 2012, 2022; Averyanov et al. 2014; Li et al. 2024, 2025; Xu et al. 2025; Qiu et al. 2025; He et al. 2025). Most of these new species are mainly discovered in the HHM. It is worth mentioning that divergence time analysis has revealed a close relationship between the diversity of *Sinocrassula* and the unique climate and geological history of HHM (Ding et al. 2017; Sun et al. 2017; Zhao et al. 2025). In our previous study, we employed genome‐skimming sequencing, transcriptome sequencing, and flow cytometry for the first time to conduct a comprehensive and systematic investigation of the interspecific relationships within *Sinocrassula*, as well as its origin and diversification (Zhao et al. 2025). The results demonstrated that *Sinocrassula* not only exhibits rapid radiation but is also susceptible to biological factors such as hybridization and incomplete lineage sorting. Interestingly, we also identified two putative new species of *Sinocrassula* that always formed two independent lineages.

In this study, using a combined dataset of chloroplast and nuclear genes alongside morphological evidence, we confirm two putative new species, which are distinct from any known species of *Sinocrassula*, and name them as *S. crassifolia* Chao Chen, Jing Zhao & J. Guan Wang and *S*. *yongshengensis* Chao Chen & J. Guan Wang. *S*. *yongshengensis* is distributed only in Yongsheng County in northwestern Yunnan Province, while *S. crassifolia* is found exclusively in Qiaojia County in northeastern Yunnan Province. The discovery of *S. crassifolia* originated when the first author of this study conducted supplementary field survey of *Cheilanthes qiaojiaensis* (Chu et al. 2021). Over the subsequent four years, the authors of this study carried out two additional observational and field collection expeditions for those two new species.

## 2 Materials and Methods

### 2.1 Morphological Measures and Observations

Plants were collected from the field during September 2020 to May 2025. Fresh plant materials were grown in the greenhouse of Yunnan University. The specimens were deposited in the herbaria YUKU. Herbarium abbreviations and acronyms follow Thiers (2024). The descriptions and characteristics of the new species were based on living plants, more field surveying, and populations that may alter the current morphological boundaries established in the description. Photographs were taken using a camera (Nikon, Japan) and an SMZ1270 stereo microscope (Nikon, Japan) from living plants. Morphological characteristics were measured using ImageJ based on at least five individuals from each of the populations.

### 2.2 DNA Extraction, Sequencing, and Assembly

Total DNA was extracted following the pipeline provided in the TIANGEN plant genomic DNA extraction kit (TIANGEN Biotech., Beijing, China). Each sample yielded approximately 1.5 μg of DNA, which served as the starting material for the preparation of DNA libraries. The short‐read sequencing (2 × 150 bp) was performed on the Illumina Nova 6000 platform by Biomaker Technology Co. Ltd. (Beijing, China). About 2 Gbp of raw data were obtained for each sample. Raw reads underwent filtration in Fastp v0.23.1 (Chen et al. 2018) to procure high‐quality clean reads. High‐quality paired‐end reads were assembled into complete plastomes using GetOrganelle v1.7.5 (Jin et al. 2020), with parameters set to *R* = 30 and k = 21, 45, 65, 85, 105, 115. For the nuclear ribosomal sequences, we conducted the assembly in the GetOrganelle pipeline similar to the procedure for plastome assembly, but with the k‐mer size used in SPAdes set as 35, 85 and 115. To extract plastid genes and nuclear genes from the assemblies for the following analyses, we annotated the final assemblies in GeSeq v2.03 (Tillich et al. 2017) and BLASTn via NCBI. Annotation visually inspected and edited by hand where necessary in Geneious Prime 2019.2.1. Circular plastid genome map was drawn with OrganellarGenomeDRAW v1.3.1 (Greiner et al. 2019).

### 2.3 Sequence Alignment and Phylogenetic Analyses

To determine the phylogenetic position of the new species and integrate all available sequence data. Two datasets were generated: (a) a matrix including four plastid DNA regions (*mat*K, *psb*A‐*trn*H, *rbc*L, and *trn*L‐*trn*F) and one nuclear region (ITS) from 53 taxa (Table S1) which were downloaded from GenBank and newly generated data in this study; and (b) another matrix including 76 plastid protein coding gene and 74 protein noncoding regions from 46 samples (Table S2). The phylogenetic tree of *Sinocrassula* was constructed through both Maximum Likelihood (ML) and Bayesian Inference (BI) methods. Each gene was aligned using Mafft v7.450 (set E‐INS‐i) (Katoh and Standley 2013) and following trimming in trimAI v1.3 (set ‐automated1) (Capella‐Gutierrez et al. 2009). ModelFinder (Kalyaanamoorthy et al. 2017) was employed to select the best‐fitting likelihood model for ML and BI under the corrected Akaike information criterion (AICc). ML bootstrapping was conducted using IQ‐tree v2.1.3 (Nguyen et al. 2015) with 5000 rapid bootstrap (BS) analyses followed by a search for the best‐scoring tree in a single run. BI approach implemented in MrBayes v3.2.2 (Ronquist et al. 2012). The Markov Chain Monte Carlo (MCMC) was set to 20, 000,000 generations, sampling every 1000 generations with a temperature of 0.2, for two runs with four chains. The standard deviation of splits frequencies below 0.001, and the MCMC output was examined to check for convergence and to ensure that the effective sample size (ESS) values were > 200. The trees were viewed with FigTree v1.4.3 (http://tree.bio.ed.ac.uk/software/figtree/).

## 3 Results

### 3.1 Morphological Characters

Morphologically, a detailed comparison between the new species and its morphologically most similar species is presented in Table 1.

**TABLE 1:** Morphological comparison with similar species.

Character	*Sinocrassula adpressa*	*S. crassifolia*	*S. ganluoensis*	*S. obliquifolia*	*S. indica* var. *obtusifolia*	*S. yongshengensis*
Life cycle	Perennial	Perennial	Perennial	Perennial	Perennial	Perennial
Basal leaves	Rosulate, spatulate	Rosulate, pear‐shaped or spindle‐shaped	Rosulate, orbicular	Rosulate, asymmetrically ovoid to lanceolate	Rosulate, spatulate and obtuse‐rounded	Rosulate, spatulate‐oblong
Plant surface	Glabrous	Glabrous	Glabrous	Glabrous	Glabrous	Glabrous
Stem leaves	Purple red with spots	Purple red	Green to purple red	Purple red	Gray‐green	—
Bracts	Elliptic	Lanceolate	Lanceolate	Lanceolate	Resembling distal stem leaves	Spatulate or ovate‐oblong
Inflorescences	8–20 cm	3–10 cm	5–11.5 cm	10–18 cm	4–8 cm	4.5–15 cm
Flowers color	Reddish purple	Pinkish white	Reddish purple	Reddish purple	Reddish	White to green
Petals	Central protrusion	Without protrusion	Without protrusion	Without protrusion	Without protrusion	Upper middle with ridged protrusion
Nectar scales	Rectangular	Reniform	Quadrate	Rectangular	Nearly ligulate	Rectangular
Nectar scales size	0.5–0.7 × 0.3–0.5 mm	0.5–1.0 × 0.4–0.5 mm	0.5 × 0.9 mm	0.3–0.5 × 0.2–0.3 mm	0.5 × 0.6 mm	0.4–0.7 × 0.3–0.5 mm
Phenology	Sep.–Nov.	Apr.–Jun.	Jul.–Oct.	Jun.–Oct.	Jul.–Oct.	Aug.–Oct.

### 3.2 Characteristics of the Complete Plastome

**FIGURE 1:**
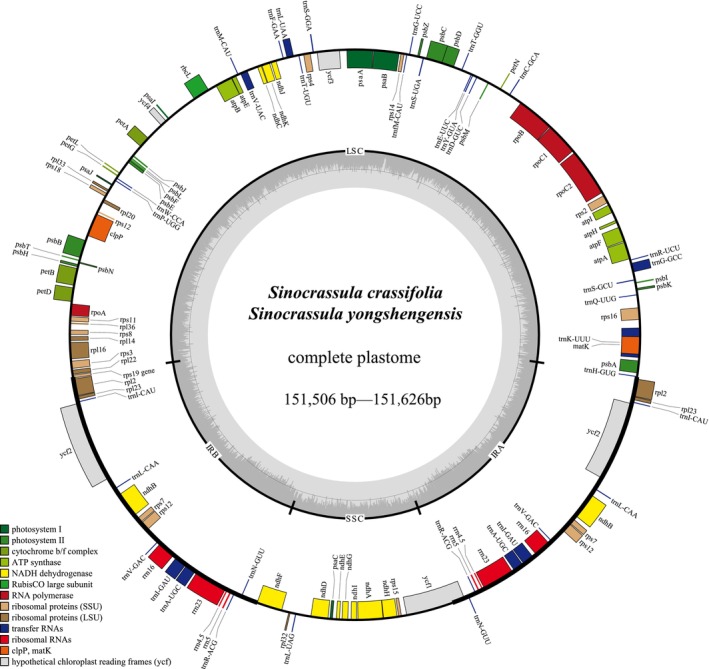
The plastome annotation map of *Sinocrassula crassifolia* and *S*. *yongshengensis*. The darker gray in the inner circle corresponds to GC content. The IRA and IRB (two inverted repeating regions); LSC (large single‐copy region); and SSC (small single‐copy region) are indicated outside of GC content.

The complete plastome of the two new species displayed the typical quadripartite structure containing 130 genes (Figure 1). Plastomes sizes of those two new species were range from 151,506 bp in *Sinocrassula crassifolia* (voucher: YUS14579) to 151,581 bp in *S*. *yongshengensis*, consisting of a large single copy (LSC; 82,922 bp–82,976 bp) region, a small single copy (SSC; 16,841 bp–16,885 bp) region, and two inverted repeated (IR; 25,860 bp–25,873 bp) regions (Figure 1; Table S2). The overall Guanine‐Cytosine (GC) content was the same across samples (37.70%) (Table S2).

The 130 plastid genes had identical order, comprising 85 protein‐coding genes, 37 transfer RNA (tRNA) genes, eight ribosomal RNA (rRNA) genes (Figure 1). The 5‐end exon of the *rps*12 gene was located in the LSC, and the intron and 3‐end exon of the gene were situated in the IR region (Figure 1).

### 3.3 Phylogenetic Relationships

**FIGURE 2:**
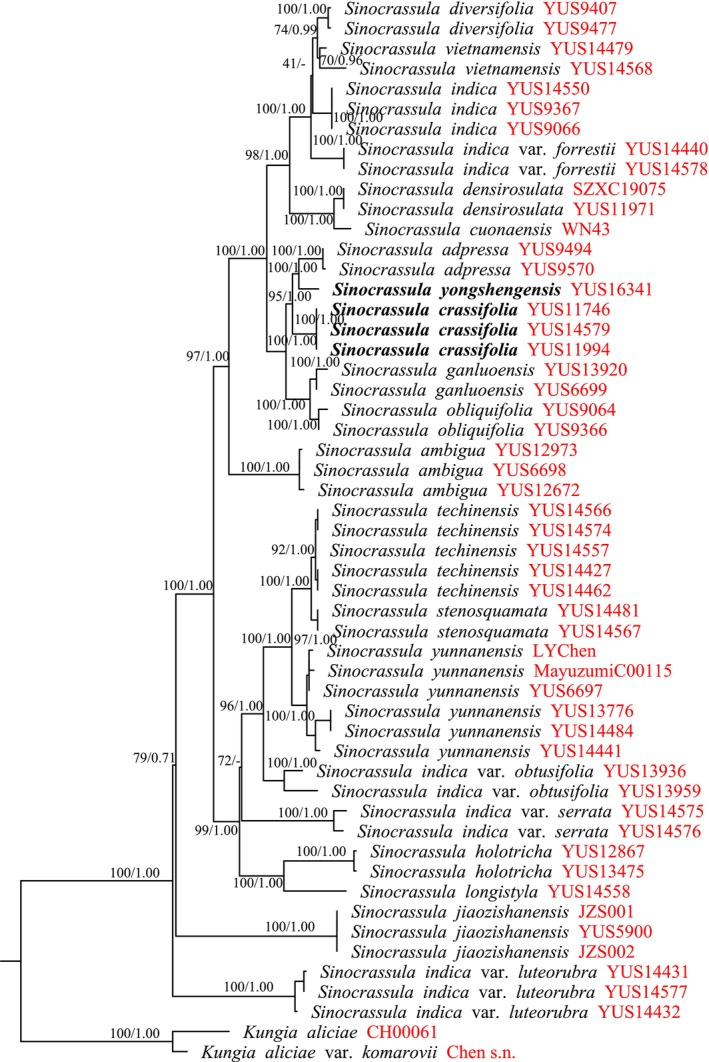
ML phylogenetic tree of *Sinocrassula* based on four plastid DNA regions (*mat*K, *psb*A‐*trn*H, *rbc*L, and *trn*L‐*trn*F) and one nuclear region (ITS). Maximum likelihood bootstrap support (ML‐BS) and Bayesian inference posterior probability (BI‐PP) are given above the branches.

**FIGURE 3:**
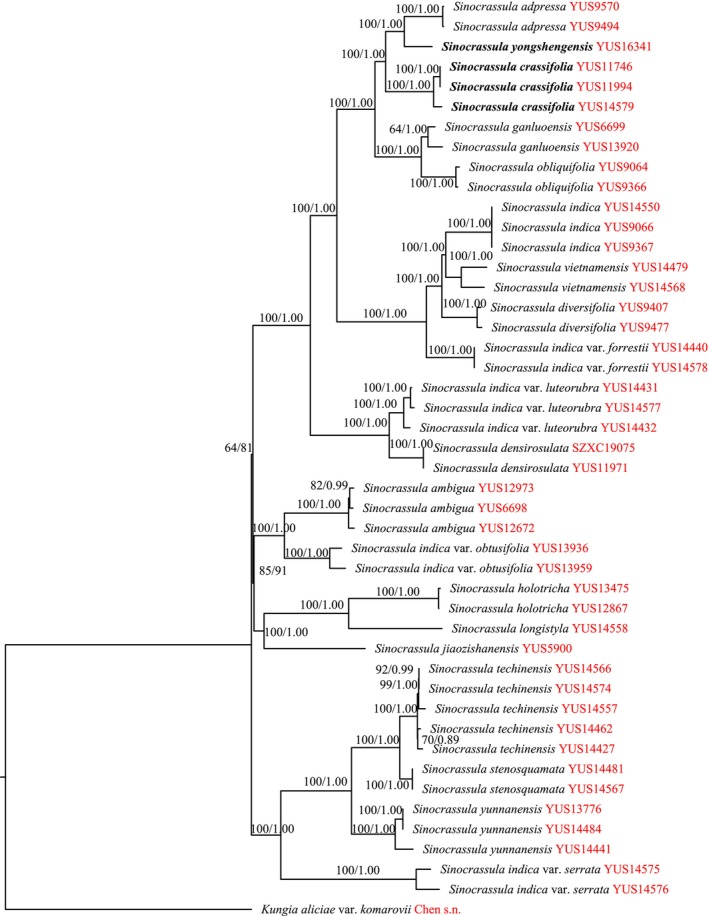
ML phylogenetic tree of *Sinocrassula* based on 150 plastid regions. Maximum likelihood bootstrap support (ML‐BS) and Bayesian inference posterior probability (BI‐PP) are given above the branches.

The datasets characteristics and best‐fitting likelihood models for the analyses are presented in Table S3. The alignment of four plastid markers and one nuclear marker was 5020 characters long, of which 4516 sites were identical, and 371 characters were parsimony‐informative. The concatenated 150 plastid regions datasets were 136,245 bp in length. The K3Pu + F + I + G4 and GTR + F + I + G4 were the best models of evolution in ML and BI analyses, respectively. The ML and BI analyses generated trees based on the same dataset with similar topologies without strongly supported conflicts. The ML trees are shown in Figures 2 and 3.

In our phylogeny, *Kungia* was strongly supported as sister to the *Sinocrassula* (Figures 2 and 3). Within the *Sinocrassula*, *S. crassifolia* was sister to *S*. *yongshengensis* and *S*. *adpressa*. Together, these three species were strongly supported as sister to *S*. *ganluoensis* + *S*. *obliquifolia*. All these relationships were strongly supported (MLBS ≥ 95%; BIPP = 1.00) (Figures 2 and 3).

## 4 Discussion

### 4.1 Morphological and Molecular Evolution

**FIGURE 4:**
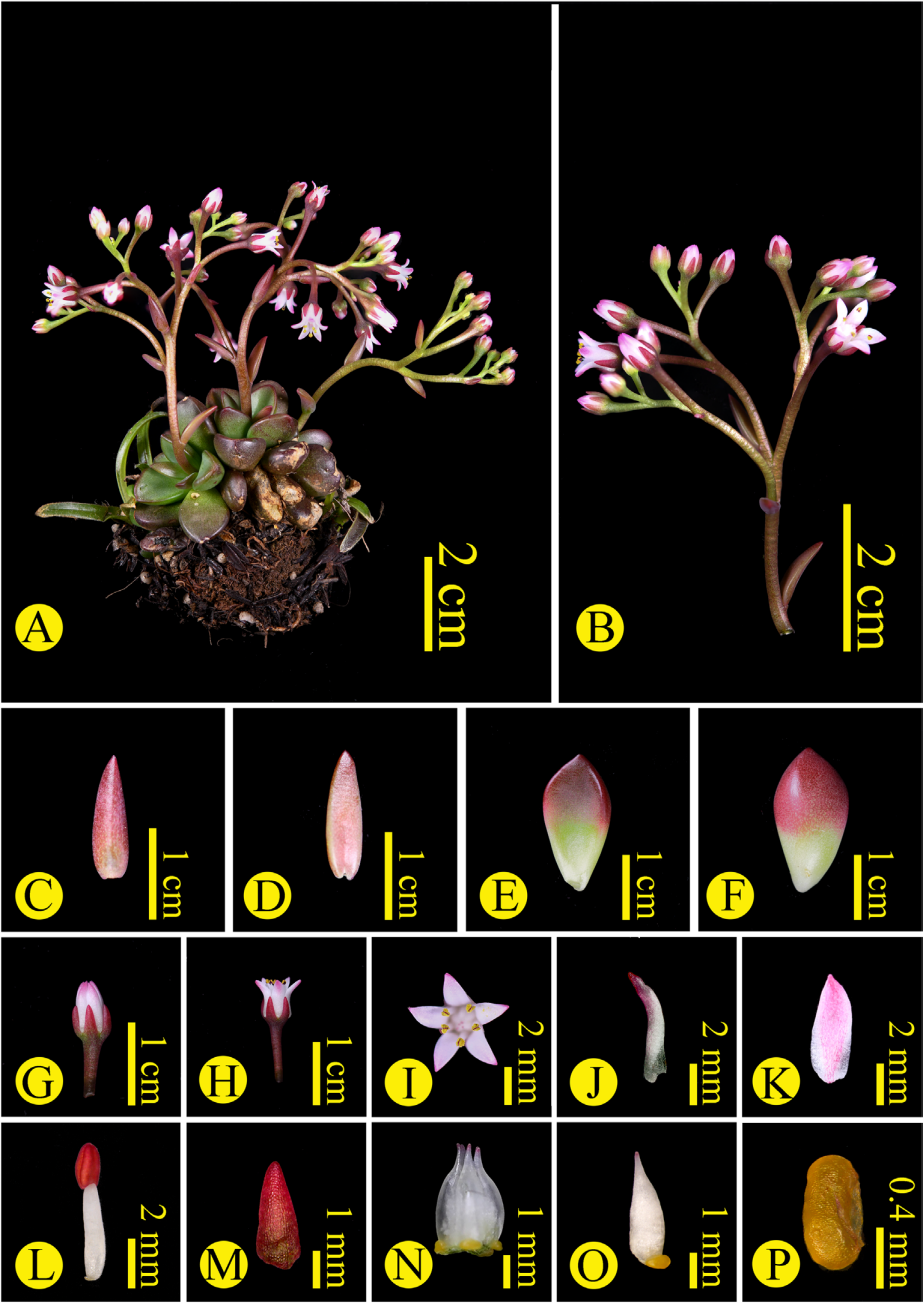
*Sinocrassula crassifolia*. (A) Habit, (B) Inflorescence, (C, D) Bracts, (E, F) Basal leaves, (G, H, I) Flower, (J, K) Petal, (L) Stamen, (M) Sepal, (N, O) Carpels, (P) Nectar scale.

**FIGURE 5:**
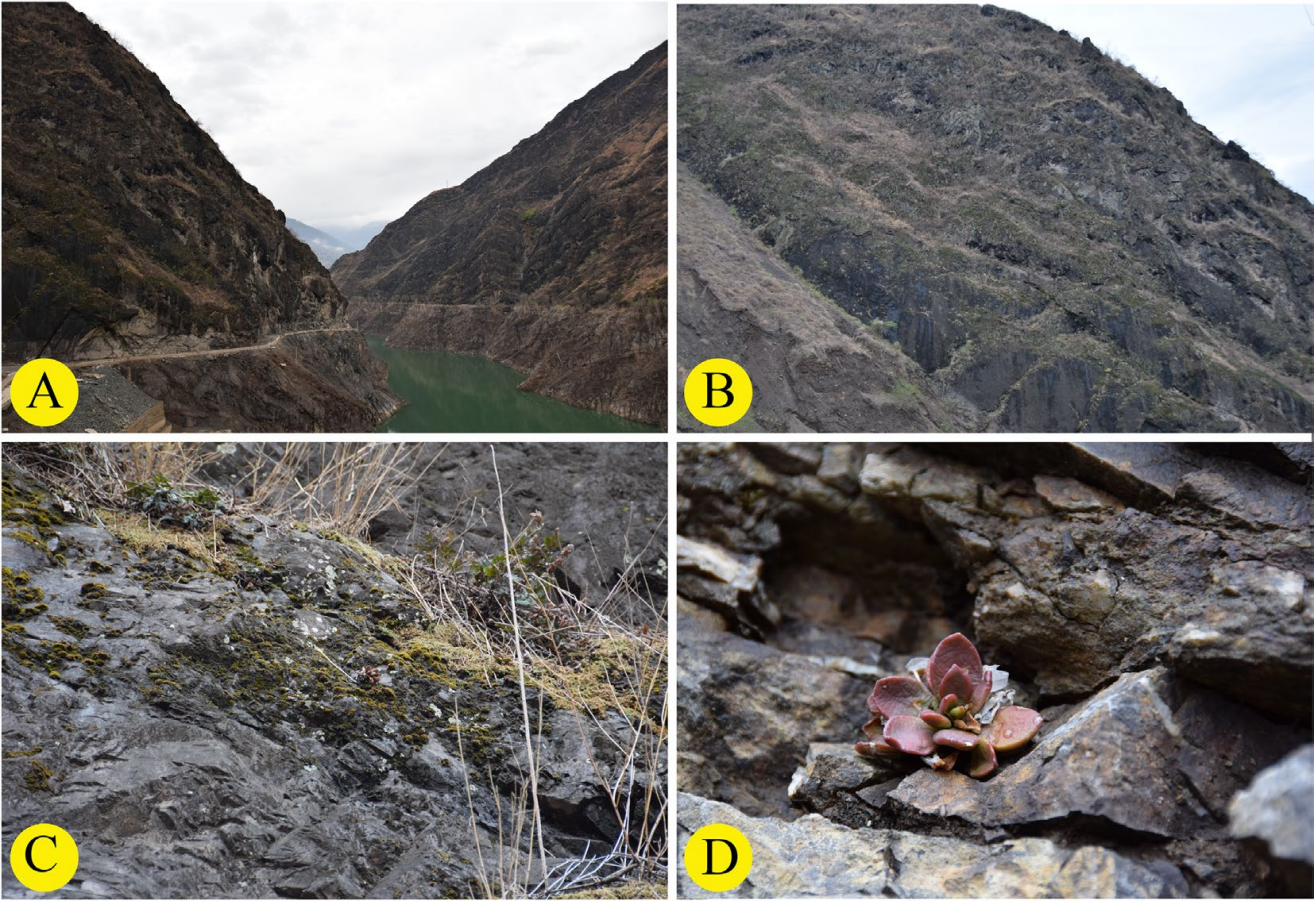
Field information of *Sinocrassula crassifolia*. (A, B, C) Habitat, (D) Habit.

**FIGURE 6:**
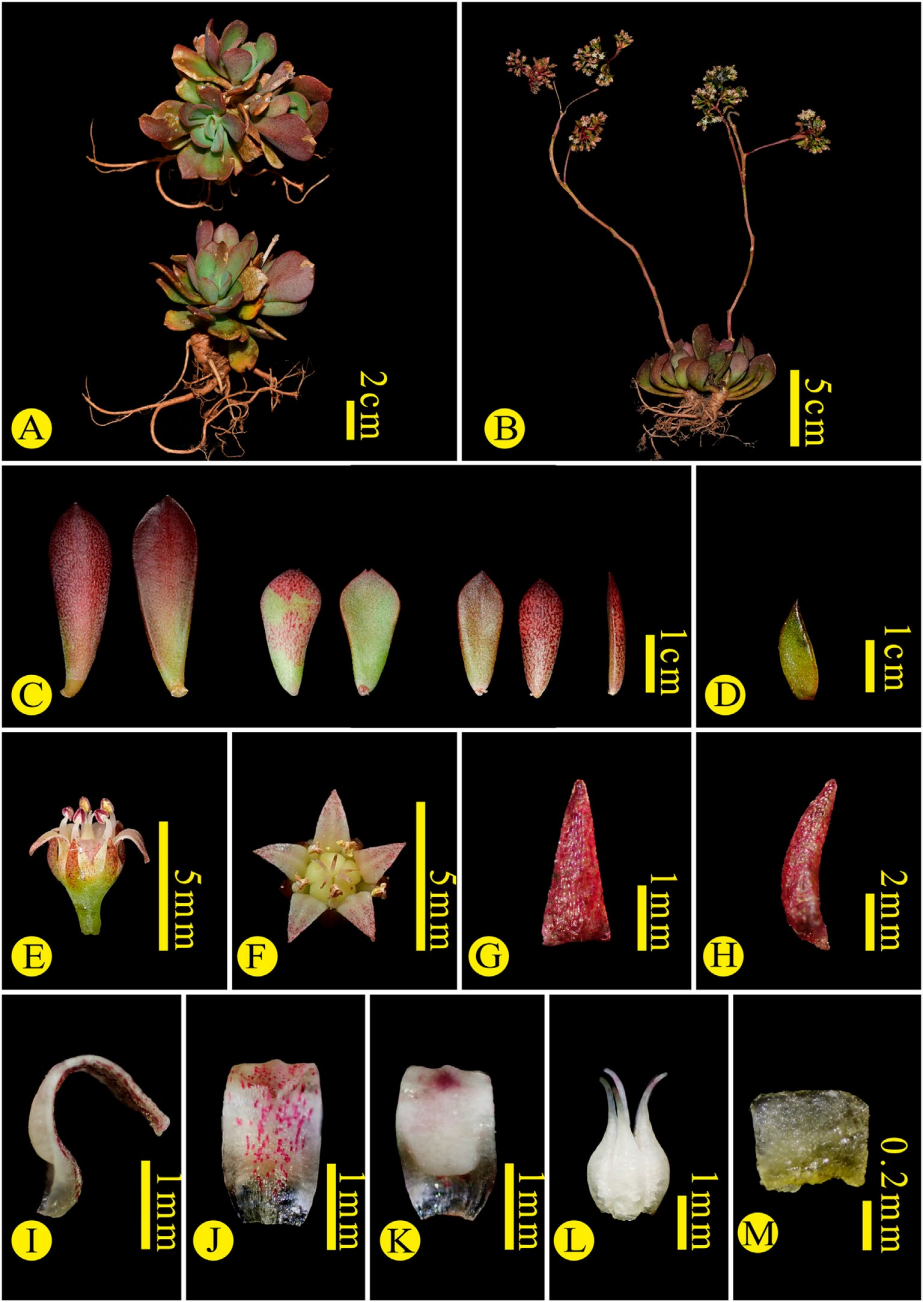
*Sinocrassula yongshengensis*. (A, B) Habit, (C) Basal leaves, (D) Bracts, (E, F) Flower, (G, H) Sepal, (I, J, K) Petals, (L) Carpels, (M) Nectar scale.

**FIGURE 7:**
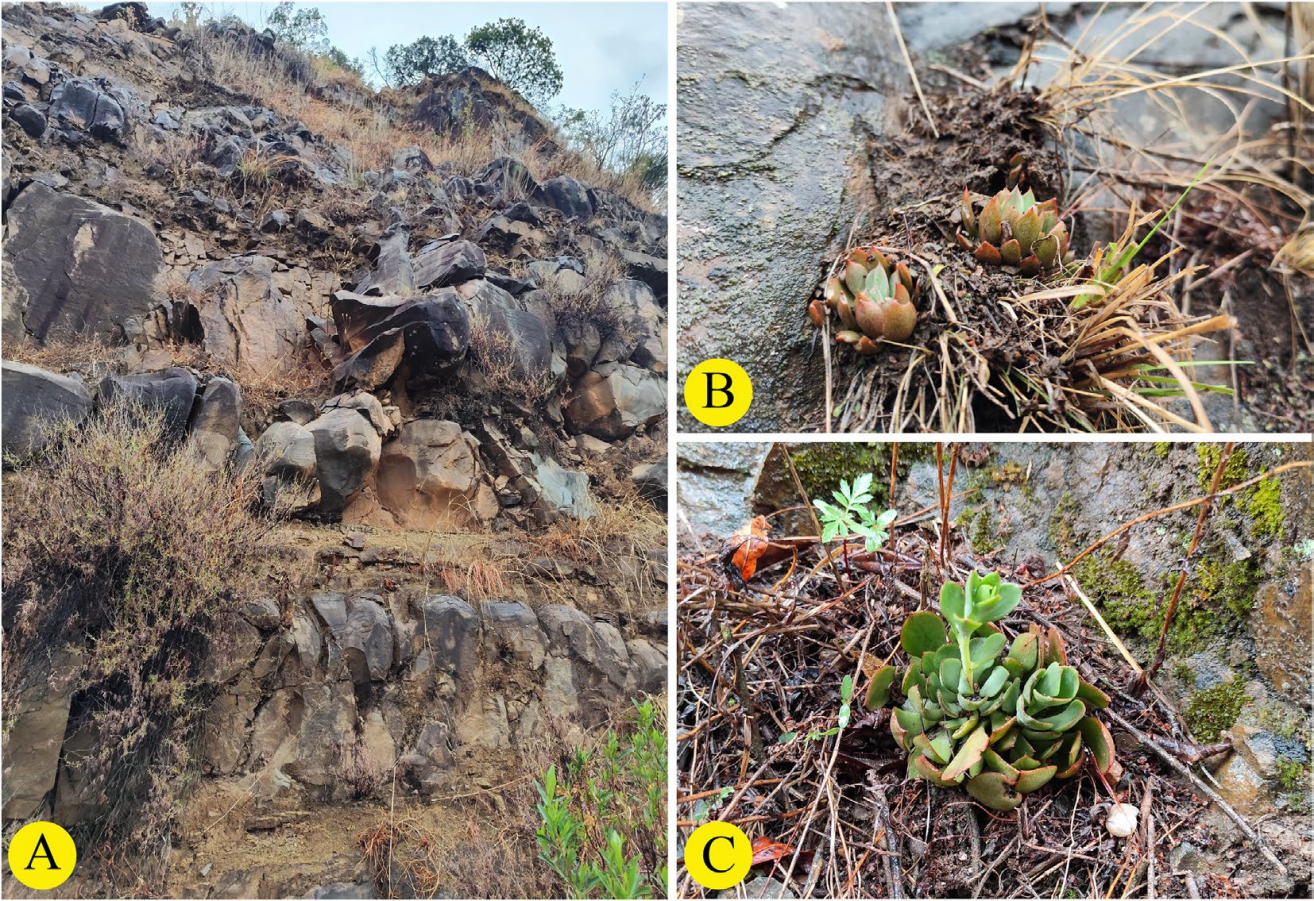
Field information of *Sinocrassula yongshengensis*. (A) Habitat, (B, C) Habit.

By combining both molecular and morphological evidence, we confirm that *Sinocrassula crassifolia* (Figures 4 and 5) and *S. yongshengensis* (Figures 6 and 7) are succulent plants endemic to southwestern China. The discovery of these two species further enriches the diversity of the genus *Sinocrassula* in China and provides important plant resource for subsequent research on speciation within this genus (Fu and Ohba 2001; Zhao et al. 2025). However, we found that the patterns of morphological evolution and molecular evolution within *Sinocrassula* are not congruent. For example, the presence of hairs on plant surfaces has so far only been observed in *S*. *holotricha* and *S. yunnanensis* (Fu and Ohba 2001; Xu et al. 2025), yet these two species are distantly related and are not sister species based on different datasets (Figures 2 and 3). Furthermore, two different molecular datasets both showed that *S. ambigua* , *S*. *jiaozishanensis*, and *S*. *stenosquamata*, which lack a rosette habit (Fu and Ohba 2001; Wang et al. 2012, 2022), are not sister species and belong to different clades (Figures 2 and 3). In contrast, *S*. *techinensis* and *S*. *stenosquamata* are not only similar in morphology but are also supported as sister species by molecular phylogenetic data (Fu and Ohba 2001; Wang et al. 2012; Figures 2 and 3). This indicated that the evolutionary history of *Sinocrassula* includes both congruent and incongruent patterns between morphology and molecules. Notably, although morphological characters can clearly distinguish *S*. *techinensis* and *S*. *stenosquamata*, the molecular phylogeny based on four chloroplast genes and one nuclear gene shows short branch lengths between the two species (Figure 2), whereas the plastome‐based data yield longer branch lengths (Figure 3). This implied that plastome data has greater advantages in resolving species boundaries among closely related species within *Sinocrassula*. Therefore, we urge that future studies on *Sinocrassula* should not only make use of omics data whenever possible, but also incorporate other genomic sequences from mitochondrial genes, nuclear genes, SNPs, and other markers for phylogenetic‐based analyses.

### 4.2 Taxonomy Implications

The species diversity of the genus *Sinocrassula* may far exceed our current understanding. The drivers of this diversity mainly include biotic and abiotic factors (You et al. 2024; Zhao et al. 2026). Among the abiotic factors, we hypothesize that the unique geological history and climate change of the HHM region have played a major role in the diversification of *Sinocrassula* species. In our previous field surveys and divergence time estimation studies, we found that *Sinocrassula* is mainly distributed in the HMM region and began to diversify during the Neogene period (Zhao et al. 2025). This region has unique geological landforms due to plate collision, and combined with monsoon variations, the climatic environment is highly heterogeneous (Sun et al. 2017; Ding et al. 2020). The main biotic factors are mostly related to hybridization and incomplete lineage sorting resulting from rapid radiation. Based on different datasets, we found that the ancestral branch lengths of *Sinocrassula* are relatively short (Figures 2 and 3), suggesting that species of the lineage may have undergone rapid diversification within an extremely short time, leading to an explosive emergence of numerous species. Moreover, the incongruent phylogenetic relationships among different datasets also imply that extensive natural hybridization may exist among *Sinocrassula* (Figures 2 and 3). Therefore, there may still be many new species and cryptic species within *Sinocrassula* that require extensive field investigations and comprehensive, systematic research.

### 4.3 The Protection of *Sinocrassula*

This study found that the habitats of *Sinocrassula crassifolia* and *Cheilanthes qiaojiaensis* (Chu et al. 2021), which were previously described, have been severely disturbed by human activities due to the construction of local hydropower and water conservancy projects. Although plants of the genus *Sinocrassula* generally possess strong drought tolerance, the survival of these species is now threatened. Apart from *S*. *techinensis*, which has been listed as a protected plant, no other *Sinocrassula* species are currently under protection (IUCN 2024; Gao et al. 2025). Although China is a center of diversity for the genus *Sinocrassula*, awareness and understanding of the need to conserve these species remain lacking. We here advocate for further extensive population surveys and conservation efforts during future field investigations of *Sinocrassula* species. Furthermore, since many *Sinocrassula* species are narrowly distributed endemics (Wang et al. 2012, 2022; Li et al. 2024, 2025; Xu et al. 2025; Qiu et al. 2025; He et al. 2025), additional measures such as in situ conservation, ex situ conservation, and artificial propagation should be implemented to preserve the genetic diversity of the genus as much as possible. Combined with the IUCN Red List categories and criteria for species conservation, this will lay a foundation for the future utilization and development of the ornamental and medicinal value for some species of *Sinocrassula*.

## 5 Conclusion and Future Perspectives

This study reports two new species of the genus *Sinocrassula*, which are endemic to Yunnan Province, China. Through morphological comparisons and molecular phylogenetic research, we found that these two species are closely related to *S*. *adpressa* (He et al. 2025) (Figures 2 and 3). Analyses based on different datasets revealed varying phylogenetic relationships within the genus *Sinocrassula*, which may be attributed to factors such as rapid radiation, hybridization, and incomplete lineage sorting (Zhao et al. 2025). The discovery of these new species also highlights the diversity of *Sinocrassula* in China and lays a foundation for further exploration of their medicinal and ornamental value. Given that most *Sinocrassula* species are distributed in the HHM region, the unique geological history and complex climatic environment of this area may have played a significant role in the speciation of *Sinocrassula* (Zhao et al. 2025). We advocate for future research to strengthen comprehensive and systematic studies on the driving factors of *Sinocrassula* diversification, including topography, climate, monsoons, and other relevant elements.

## 6 Taxonomic Treatment

***Sinocrassula crassifolia*** Chao Chen, Jing Zhao & J. Guan Wang, sp. *nov* (Figures 4 and 5).

**Type:** CHINA. Yunnan Province: Zhaotong City, Qiaojia County, Jintang Town, 26°47′26.5″ N, 103°0′46.2″ E, elev. ca. 800 m, on xeric limestone rocks, 25 Dec. 2020, *Jing Zhao* et al. *YUS11746* (holotype YUKU!, isotypes YUKU!).

**Diagnosis:** Morphologically, *Sinocrassula crassifolia* resembles *S. indica* var. *obtusifolia*, but there are obvious differences in the basal leaves: the basal leaves of *S. crassifolia* have an acuminate apex, with the base being light green and the middle and apex being deep red, while the basal leaves of *S. indica* var. *obtusifolia* have an obtuse apex and are mostly gray‐green.

**Description:** *Plant* terrestrial or lithophytic, perennial, 4–15 cm tall, glabrous, rosette compact. *Roots* fibrous. *Basal leaves* pear‐shaped to spindle‐shaped, becoming spatulate after dehydration, apex acuminate, 0.7–1.5 × 0.5–0.75 cm, thickness about 0.6–1.0 cm, green at the base, middle to apex purple‐red. *Flowering stem* terminal, 3–10 cm long, slightly curved. *Stem leaves* alternate, lanceolate, 0.8–1.0 × 1.5 cm. *Inflorescence* corymbiform, many flowers, with pedicels. *Bracts* linear or narrowly triangular. *Pedicels* 2.8–4.2 mm long. *Flowers* small, ca. 3.0–5.0 mm in diam. *Sepals* narrowly triangular, deep red, densely papillate, 2.0–2.5 × 0.8–1.5 mm. *Petals* white to pink, oblong to ovate‐orbicular, apex obtuse or acuminate, 2.0–4.0 × 0.5–1.5 mm. *Stamens* slightly longer than the petals, 3.5–5.0 mm, filaments white, gradually widening at the base, anthers purple‐red, dehiscing longitudinally, pollen golden yellow. *Nectar scales* yellow, reniform, 0.5–1.0 × 0.4–0.5 mm. *Carpels* 5, ovate‐lanceolate, about 4.5 mm. *Flowering* period from April to June.

**Additional specimens examined (paratypes):** Yaoshan Town, Qiaojia County, Yunnan Province, CHINA, alt. 750 m, 23 Dec. 2023, *Jie Zhou* et al. *YUS‐14579* (YUKU!).

**Conservation status:** Only two populations of *Sinocrassula crassifolia* are currently known from Jintang and Yaoshan Town, Yunnan Province, China. Based on current evidence and following International Union for Conservation of Nature (IUCN) Categories and Criteria (IUCN 2024), its restricted geographic range and small population size suggest that the species should be assessed as “Data Deficient (DD)”.

**Geographical distribution:** *Sinocrassula crassifolia* is only found in Qiaojia County, Yunnan Province, and may represent an endemic species in Northeastern Yunnan.

**Ecology:** *Sinocrassula crassifolia* was observed to grow on xeric limestone rocks at an elevation of 750–800 m.

**Etymology:** The species epithet “*crassifolia*” refers to this species having thick basal leaves, a morphological feature that distinguishes it from other species of *Sinocrassula*. Its Chinese name is suggested as “厚叶石莲 (hou ye shi lian)”.

***Sinocrassula yongshengensis*** Chao Chen & J. Guan Wang, sp. *nov* (Figures 6 and 7).

**Type:** CHINA. Yunnan Province: Lijiang City, Yongsheng County, Shunzhou Town. 26°38′51.3096″ N, 100°37′50.232″ E, elev. ca. 2150 m, on rocks in shade. 1 July 2021, *Chao Chen* et al. *YUS‐16341* (holotype YUKU!, isotypes YUKU!).

**Diagnosis:** Morphologically, *Sinocrassula yongshengensis* is similar to *S. indica* var. *obtusifolia*. The basal leaves of *S. indica* var. *obtusifolia* and *S. yongshengensis* are both spatulate, but the apex of the leaves of *S. indica* var. *obtusifolia* is obtuse, with both bracts and petals being red, while the bracts of *S. yongshengensis* are green, with petals that are white at the base and have purple‐red spots at the apex.

**Description:** *Plant* terrestrial or lithophytic, perennial, 5–20 cm tall, glabrous, rosette. *Roots* fibrous. *Basal leaves* spatulate‐oblong, apex acuminate, 1.2–3.5 × 0.3–1.5 cm, green at the base, slightly red at the apex with reddish purple stripes. *Flowering stem* terminal, 4.5–15 cm long, slightly curved, branched or unbranched. *Inflorescence* corymbiform, many flowers. *Bracts* spatulate or ovate‐oblong, green, 3–4 mm in diameter, with pedicels. *Pedicels* 2.8–4.2 mm long. *Flowers* small, ca. 5.0–8.0 mm in diam. *Sepals* triangular, purple‐red, 1.2–2.5 × 0.6–1.1 mm. *Petals* white at the base, apex with purple‐red spots, upper middle with ridged protrusion and swollen at the base, narrow triangular, apex acuminate, 2.5–3.5 × 1.0–1.5 mm. *Stamens* slightly shorter than the petals, 1.7–3.2 mm, filaments white, gradually widening at the base, anthers oblong, pink to red, 0.4–0.9 mm long. *Nectar scales* white, nearly rectangular, 0.4–0.7 × 0.3–0.5 mm. *Carpels* 5, lanceolate, 1.7–2.6 mm. *Flowering* period from August to October.

**Conservation status:** Only one population of *Sinocrassula yongshengensis* is currently known from Shunzhou Town, Yongsheng County, Yunnan Province, China. Based on current evidence and following International Union for Conservation of Nature (IUCN) Categories and Criteria (IUCN 2024), its restricted geographic range and small population size suggest that the species should be assessed as “Data Deficient (DD)”.

**Geographical distribution:** Currently, *Sinocrassula yongshengensis* is only found in Yongsheng County, Yunnan Province based on our current knowledge and may represent a species endemic to Northwestern Yunnan.

**Ecology:** *Sinocrassula yongshengensis* is observed to grow in the crevices of rocks at elevations between 2100 and 2300 m.

**Etymology:** The epithet “*yongshengensis*” refers to Yongsheng County where the species was discovered. Its Chinese name is suggested as “永胜石莲 (yong sheng shi lian)”.

## Author Contributions

**Jing Zhao:** data curation (lead), formal analysis (lead), investigation (lead), methodology (lead), software (lead), validation (lead), visualization (lead), writing – original draft (lead), writing – review and editing (lead). **Ling‐Nan Wei:** data curation (lead), formal analysis (lead), investigation (lead), methodology (lead), software (lead), validation (lead), visualization (lead), writing – original draft (lead), writing – review and editing (lead). **Jie Zhou:** investigation (lead), resources (lead), writing – original draft (supporting). **Jian‐Rong Zhang:** investigation (supporting), resources (supporting). **Xin‐Mao Zhou:** investigation (supporting), supervision (supporting). **Zhao‐Rong He:** investigation (supporting), resources (supporting). **Chao Chen:** funding acquisition (equal), investigation (lead), resources (lead), supervision (supporting), writing – original draft (lead), writing – review and editing (lead). **Jia‐Guan Wang:** conceptualization (lead), funding acquisition (equal), investigation (supporting), project administration (lead), visualization (supporting), writing – original draft (lead), writing – review and editing (lead).

## Funding

This work was supported by Funding for Undergraduate University‐Reserve‐Research (URR) Cultivation Program, School of Life Sciences, Yunnan University and Yunnan Key Laboratory of Forest Ecosystem Stability and Global Change, Xishuangbanna Tropical Botanical Garden (Grant No. 202449CE340025).

## Conflicts of Interest

The authors declare no conflicts of interest.

## Supporting information


**Table S1:** Voucher and GenBank accession information of each species used. A dash (—) indicates missing data.
**Table S2:** List of plastomes used in this study.
**Table S3:** Best‐fitting models and characteristics in different datasets in this study.

## Data Availability

The DNA sequences generated in this study have been deposited in the National Center for Biotechnology Information (NCBI) database. The accession numbers and the information on the voucher specimens are available in Tables S1 and S2. Voucher specimens of the new species are deposited at YUKU.
